# Comparison of Phase Estimation Methods for Quantitative Susceptibility Mapping Using a Rotating-Tube Phantom

**DOI:** 10.1155/2021/1898461

**Published:** 2021-11-24

**Authors:** Kathryn E. Keenan, Ben P. Berman, Slávka Rýger, Stephen E. Russek, Wen-Tung Wang, John A. Butman, Dzung L. Pham, Joseph Dagher

**Affiliations:** ^1^National Institute of Standards and Technology, Physical Measurement Laboratory, 325 Broadway, Boulder, CO 80305, USA; ^2^The MITRE Corporation, 7515 Colshire Dr, McLean, VA 22102, USA; ^3^Henry M. Jackson Foundation, 10 Center Drive, Bethesda, MD 20892, USA; ^4^Clinical Center, National Institutes of Health, 10 Center Drive, Bethesda, MD 20814, USA

## Abstract

Quantitative Susceptibility Mapping (QSM) is an MRI tool with the potential to reveal pathological changes from magnetic susceptibility measurements. Before phase data can be used to recover susceptibility (Δ*χ*), the QSM process begins with two steps: data acquisition and phase estimation. We assess the performance of these steps, when applied without user intervention, on several variations of a phantom imaging task. We used a rotating-tube phantom with five tubes ranging from Δ*χ*=0.05 ppm to Δ*χ*=0.336 ppm. MRI data was acquired at nine angles of rotation for four different pulse sequences. The images were processed by 10 phase estimation algorithms including Laplacian, region-growing, branch-cut, temporal unwrapping, and maximum-likelihood methods, resulting in approximately 90 different combinations of data acquisition and phase estimation methods. We analyzed errors between measured and expected phases using the probability mass function and Cumulative Distribution Function. Repeatable acquisition and estimation methods were identified based on the probability of relative phase errors. For single-echo GRE and segmented EPI sequences, a region-growing method was most reliable with Pr (relative error <0.1) = 0.95 and 0.90, respectively. For multiecho sequences, a maximum-likelihood method was most reliable with Pr (relative error <0.1) = 0.97. The most repeatable multiecho methods outperformed the most repeatable single-echo methods. We found a wide range of repeatability and reproducibility for off-the-shelf MRI acquisition and phase estimation approaches, and this variability may prevent the techniques from being widely integrated in clinical workflows. The error was dominated in many cases by spatially discontinuous phase unwrapping errors. Any postprocessing applied on erroneous phase estimates, such as QSM's background field removal and dipole inversion, would suffer from error propagation. Our paradigm identifies methods that yield consistent and accurate phase estimates that would ultimately yield consistent and accurate Δ*χ* estimates.

## 1. Introduction

Quantitative Susceptibility Mapping (QSM)[1–3] is a method to estimate magnetic susceptibility of tissue from the phase of the magnetic resonance (MR) signal. QSM has potential clinical utility for characterizing neurological diseases [4–6], blood oxygen content [7], iron overload in the heart and liver [8], and quantitative tracking of contrast agent bolus perfusion [9, 10].

Repeatability and reproducibility of QSM have been assessed in phantoms and human subjects using different scanners, magnetic field strengths, and data processing methods. While some studies report high repeatability [11–18], both *in vivo* and in phantoms, recent *in vivo* studies report lower reproducibility across MRI scanners with the same data processing method [19] and across QSM algorithms using the same input data [20]. These conflicting results limit the clinical adoption of QSM.

A typical QSM process requires four steps: data acquisition (Step 1), phase estimation (Step 2), background field removal (Step 3), and magnetic susceptibility reconstruction (Step 4). Recently proposed methods combine Steps 2–4 into fewer steps [21].

In its standardization efforts, the QSM community has actively evaluated competing methods [17, 22, 23], in particular for methods in Steps 3 and 4 of the process [20]. However, the selection of the “best” QSM method is difficult for various reasons: (a) the appropriate definition of a quality metric, for example, accuracy versus repeatability; (b) competing quality metrics that favor different algorithms [20]; (c) the lack of a gold standard *in vivo*; (d) algorithm performance depending on imaging application (*in vivo* versus phantoms); and (e) the large number of methods for each QSM step, which would render any exhaustive validation effort to be combinatorial and quickly untenable.

We present an experimental setup that allows for an exhaustive quantitative analysis of all four QSM steps. This framework uses a rotating-tube phantom design introduced in the work of Erdevig et al. [24], which uses tubes that rotate, within a background solution, relative to the main magnetic field, *B*_0._ The design enables the analysis of MRI data obtained in objects at any orientation, using common QSM techniques. The closed-form theoretical relationship between the magnetic field and magnetic susceptibility in the sample allows for mapping the magnetic field to magnetic susceptibility without having to solve the dipole inversion problem [25].

Our contributions include (a) a framework for evaluation of repeatability and reproducibility of QSM algorithms and (b) rigorous analysis of common methods in Steps 1 and 2 of the QSM process. We focus on the performance of QSM Steps 1 and 2 for the following reasons:

- There exist a large number of methods in each of the four QSM steps. We replicate here approximately 90 different combinations of Steps 1 and 2 methods. If we analyze all four steps simultaneously, the resulting data set would grow combinatorially and become difficult to interpret.
- Errors introduced early in the QSM process propagate downstream and have been overlooked in validation studies. For example, Olsson et al. used only one acquisition sequence and one phase estimation algorithm [22].
- It is well understood that phase unwrapping, a common algorithm used in Step 2, is a nondeterministic polynomial-time-hard problem (in two dimensions and higher) that often relies on user intervention and careful parameter tuning. Therefore, it is important to isolate the impact of such a problem in the QSM processing methods.
- Susceptibility weighted imaging [26, 27], electrical properties tomography [28], thermometry [29], flow [30], and elastography [31] use MR phase information, and this work can inform those applications.

We analyze Steps 1 and 2 of the QSM process to understand which are sufficiently robust to be executable without user intervention and independent of scanner, sequence, and other parameter variations.

## 2. Methods

**Figure 1:**
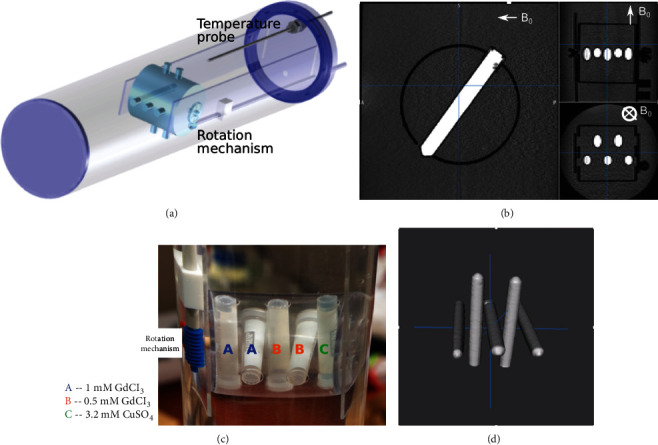
(a) 3D diagram of the rotating-tube phantom design with five smaller cylindrical samples along with temperature probe. (b) Magnitude images of the sagittal (one tube), axial, and coronal (multiple tube) views. (c) A photograph of the 5 tubes. (d) 3D rendering from MRI magnitude images.

We used a rotating-tube phantom (Figure 1(a)) to explore the reproducibility of phase estimates obtained after Steps 1 and 2. MRI data was acquired with different pulse sequences (Step 1), at nine angles, and the performance of a wide variety of phase estimation methods was compared to theory. The rotating-tube phantom was designed to take advantage of the analytical model for a long cylinder at an angle, *θ*, with respect to *B*_0_. The internal *z*-axis field offset can be derived from Maxwell's equations and is shown to be [25]

(1) $$\begin{array}{c} \delta B_{\text{in}}=\frac{\Delta \chi B_{0}}{6}\left(3\;\cos^{2}\;\theta -1\right), \end{array}$$

where  Δ*χ* is the susceptibility difference between the inside and outside of the cylinder and *χ*^2^ terms are ignored.

### 2.1. Experimental Setup

The rotating-tube phantom consists of five cylindrical, polypropylene tubes (80 mm length and 10 mm outer diameter; Nalgene cryogenic storage vials) alternated orthogonally along the central axis of a larger cylinder (610 mm length and 140 mm outer diameter) containing water (Figure 1). Each tube contains one of the following: 0.5 mM GdCl_3_, 1.0 mM GdCl_3_, or 3.2 mM CuSO_4_ (Figure 1(b)). This phantom is modifiable, and the range of susceptibility values can be focused on particular areas of interest. Here, the range of susceptibility values was selected to span those observed *in vivo* from venous blood (1.0 mM GdCl_3_) to deep grey matter structures (0.5 mM GdCl_3_) to the lower limits of MRI detection (3.2 mM CuSO_4_) [32]. A rod extends from the internal rotation gears through the phantom and outside the MRI scanner, allowing the tubes to be manually rotated. Example MR images are shown for the three primary planes (Figure 1(c)) and in a 3D rendering of the tubes (Figure 1(d)). Temperature of the water was continuously monitored via a fiber optic probe (OpSens Medical, Québec, QC, Canada).

The paramagnetic salt solution Δ*χ* values were estimated from susceptibility theory and corrected using Curie's law with the experimentally measured temperatures (21.0°C–22.0°C). Δ*χ* values were 0.336 ppm and 0.168 ppm for the 1 mM GdCl_3_ and 0.5 mM GdCl_3_, respectively, and 0.0804 ppm for the 3.2 mM CuSO_4_. Additional details on the calculation of Δ*χ* are in the Supplementary Material.

### 2.2. Data Acquisition

MR data was collected on a 3T Siemens Biograph mMR (MR-PET scanner, Syngo MR E11 software) with a 6-element torso array and 9-element spine array coil for a total of 15 elements. To assess the reproducibility of phase estimation across pulse sequences, we acquired data with four gradient echo (GRE) pulse sequences (details in Table 1):

- Single-echo GRE (SEGE): This is a commonly chosen protocol with QSM and other susceptibility-based techniques [33], wherein a single-echo time TE is measured as close to *T*_2_^*∗*^ of tissue of interest (here, the target is 60 ms for 1 mM GdCl_3_). This maximizes the phase SNR at this *T*_2_^*∗*^. To maximize the magnitude SNR at the chosen TE and TR, we set the readout bandwidth at its lowest possible value.
- Segmented Echo Planar Imaging (sEPI): A recently proposed sEPI sequence was shown to possess similar quality phase images as SEGE [34], while acquiring full brain coverage much faster than SEGE. As with SEGE, phase images were generated at a single TE at the center of the echo train.
- Multiecho GRE (MEGE): This protocol acquires multiple TEs in a single TR. The challenge with this technique is the choice of the echo spacing ΔTE and readout bandwidth BW. A short ΔTE reduces the likelihood of aliasing in the phase domain but introduces noise. A long ΔTE yields phase images with better SNR but suffers from potentially unrecoverable phase-aliasing errors. For example, to unwrap frequency offsets of ±150 Hz, ΔTE must be less than 3.33 ms. A common approach [33, 35] is to acquire data with a short ΔTE and, in order to recover SNR efficiency similar to SEGE sequences [36], acquire as many echoes as possible in a TR. However, due to hardware limitations, the readout bandwidth, BW, places a lower limit on ΔTE. We aimed to select the shortest ΔTE possible at the highest BW attainable with the MR system. This choice minimizes the likelihood of phase wrap errors, which may not be recoverable by all phase unwrapping algorithms. We elaborate on this choice further in the Discussion section.
- MAGPI: This is an MEGE sequence that uses preoptimized echo times and bandwidths selection that, when paired with a corresponding phase estimation algorithm, yields maximum-likelihood optimal phase estimates in the presence of wrapping, noise, and phase-offset errors [37, 38].

All sequences were 3D excitations of a 128 mm × 128 mm × 128 mm slab (64 slices) with sagittal slab-selection and phase encode along the *B*_0_ direction. We used anisotropic voxels to boost SNR, a common practice for QSM and Susceptibility-Weighted Imaging [26, 27, 39]. MAGPI and the sEPI sequences used alternating gradient polarity, while the other sequences did not. Autoshimming was completed prior to the first data acquisition, and then the same shim parameters were used over time, over all sequences and methods.

We assessed the effect of in-plane resolution and TR on phase estimate reproducibility with each protocol (Table 1). We also examined the reproducibility of phase estimates across nine different angles by advancing the apparatus approximately 18 degrees per turn.

### 2.3. Phase Estimation

Images generated in Steps 2–3 of QSM are commonly referred to as frequency, phase, or field maps, depending on the units of the data. We interchangeably use these names in this work depending on context, and, in our analysis, we convert all phase images to frequency via a simple scalar multiplication. Ten phase algorithms were selected to estimate the frequency offset image [37, 40–47].  Table 2 lists the methods used for each pulse sequence; multiple codes were downloaded from freely available resources (e.g., MEDI) and integrated with the pipeline.

All phase estimation methods were applied with default parameters in 3D over the entire acquisition volume and were implemented and run blinded to the theoretical solution. Apart from the MAGPI algorithm, which operates on raw k-space channel-uncombined data, all methods (including MAGPI-unopt) operated on unprocessed phase data obtained using the vendor-provided adaptive-coil-combine method. While this is “unprocessed phase data,” different vendors may apply different filters, phase corrections, or other adjustments that could influence the phase quality. Here, adaptive-coil-combine describes the algorithm used by this vendor to combine multichannel coil data [48]. Each unwrapping method used the SNR in the magnitude image to guide unwrapping orientation in the phase domain: for example, the Laplacian-based methods used this SNR to mask the entire image, while others (region-growing, GBC) masked the phase values in regions with poor SNR. SNR was measured as approximately 30 dB in water, similar to *in vivo* values ranging from 25 to 30 dB.

For MEGE sequences, the multiecho data is processed using the five following categories of algorithms:

1. Spatial phase unwrapping at each echo, followed by temporal combination of the resulting images using a weighted averaging method, that is, phase SNR-optimal [49].
2. Spatial phase unwrapping at each echo with weighted averaging (as in 1), but a 1D phase unwrapping step is used just before weighted averaging. This is meant to correct any remaining aliasing that spatial unwrapping failed to correct.
3. Direct temporal phase estimation (Slope, Division) applied in complex domain. These methods correctly unwrap the phase over time, provided the inherent frequency is less than the Nyquist frequency associated with the echo spacing.
4. Temporal phase combination (as in 3), followed by 3D spatial phase unwrapping to correct errors encountered with temporal phase estimation.
5. Maximum-likelihood-based combination of multiecho and multichannel data (MAGPI) [37]. This method solves the phase estimation problem on a voxel-by-voxel basis, without resorting to spatial averaging techniques.

All phase images are eventually converted to frequency offset (Hz) by dividing by 2*π x* phase evolution time.

### 2.4. Adjusting for Field due to Rotation of Apparatus

To accurately estimate Δ*χ* from the phase images, we need to remove the global field effects resulting from the tubes rotating in the magnetic field, as well as field effects from the apparatus itself. We call this process “frequency referencing” (FR).

The scalar magnitude of the complete field inside a voxel can be written as follows:

(2) $$\begin{array}{c} \delta B\left(r,\theta \right)=\delta B_{\Delta \chi }\left(r,\theta \right)+\delta B_{0}\left(r,\theta \right), \end{array}$$

where *r* is the spatial coordinate of the voxel and *θ* is the rotation angle of the tube relative to *B*, *δB*_Δ*χ*_(*r*, *θ*) is the field caused by magnetic susceptibility variations (such as the one due to a homogeneous cylindrical object immersed in a homogeneous sphere), and *δB*_0_(*r*, *θ*) is an unknown field offset component. We separate *δB*_0_(*r*, *θ*) into a component that varies only spatially and a component that varies only with the rotation of the apparatus:

(3) $$\begin{array}{c} \delta B_{0}\left(r,\theta \right)=\delta B_{0}^{bkg}\left(r\right)+\delta B_{0}^{rot}\left(\theta \right). \end{array}$$

At a given angle of rotation, sources of spatially varying global offsets *δB*_0_^*bkg*^(*r*) are field inhomogeneity (imperfections of magnetic field/coils), bulk magnetic susceptibility of the apparatus [50, 51], and coil phase offset [52]. As the apparatus is rotated, in the absence of “shimming” at the console, the center frequency will be shifted due to the bulk magnetic susceptibility of the entire apparatus [53, 54]. Our goal is to extract Δ*χ* by fitting *δB*(*r*, *θ*) to the angle of rotation *θ*, inside the tube. *δB*_0_^*bkg*^(*r*) is a nuisance parameter that can be easily accounted for during the fitting process by allowing for a constant shift to the cosine.

**Figure 2:**
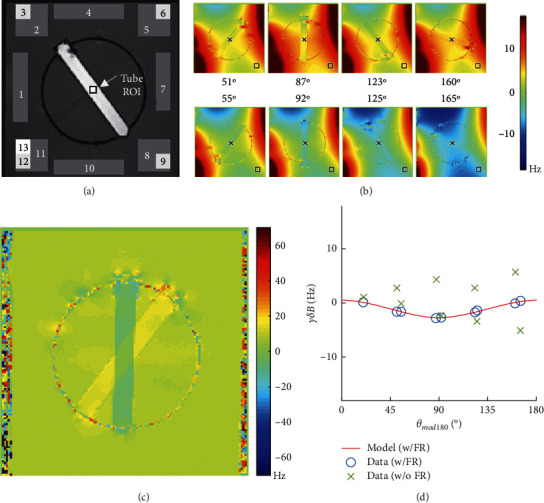
(a) An example magnitude GRE image showing both the tube ROI (small black box in center) used for the analysis and the placement of the 13 background ROIs. (b) Example frequency images in tube 3 (0.5 mM GdCl3) obtained with the single GRE sequence (1 mm, TR 45 ms) and reconstructed with MEDI.RG phase estimation method. (c) Also using SEGE + MEDI.RG, the field difference between two angles (*δ ***B**_**total**_(**r**, *θ*_2_) − *δ ***B**_**total**_(**r**, *θ*_1_)) showing that this difference is attributable to the spatially invariant component (in homogeneous areas) and a spatially variant component (in areas close to material boundaries). The spatially invariant component of the field difference is removed with frequency referencing (FR). (d) A plot of the frequency against the angle of rotation (modulo 180°) is shown for each of the data without frequency referencing (in green (*x*), data after frequency referencing using the frequency reference ROI #9 (blue circles), and the model's prediction of the frequency (solid red line).

First, we compute an estimate of *δB*_0_^*rot*^(*θ*) using the average frequency in a static region outside the “Tube + Sphere” system (Figure2(a)). The average (indicated by < >) is taken over pixels in a region *r*_out_ distant from local susceptibility effects:

(4) $$\begin{array}{c} {\langle \delta B\left(r,\theta \right)\rangle }_{r\in r_{\text{out}}}\;=\;{\langle \delta B_{0}^{bkg}\left(r\right)\rangle }_{r\in r_{\text{out}}}+\delta B_{0}^{rot}\left(\theta \right)=C_{r_{\text{out}}}+\delta B_{0}^{rot}\left(\theta \right), \end{array}$$

where we define *C*_*r*_out__ to be a variable that is only a function of the referencing region. Then, the referencing step consists of

(5) $$\begin{array}{c} \delta B_{\text{ref}}\left(r,\theta \right)\equiv \delta B\left(r,\theta \right)-{\langle \delta B\left(r,\theta \right)\rangle }_{r\in r_{\text{out}}}=\delta B_{\Delta \chi }\left(r,\theta \right)+\left(\;\delta B_{0}^{bkg}\left(r\right)-\;C_{r_{\text{out}}}\right), \end{array}$$

thus removing *δB*_0_^*rot*^(*θ*). Our goal is to use the referenced field, *δB*_ref_(*r*, *θ*), at every pixel in the tube center, *r* = *r*_in_, to fit the field variation to the angle of rotation *θ*. The only component that varies with *θ* is the first term on right side of equation (5). The estimation step is a simple fit with respect to *θ*, along with an arbitrary shift for the constant: *c* =  *δB*_0_^*bkg*^(*r*) −  *C*_*r*_out__. For the case of a cylinder (equation (1)), the estimate can be obtained by solving

(6) $$\begin{array}{c} \left(\hat{\Delta \chi }\left(r_{in}\right),c^{*}\right)=\underset{\Delta \chi ,c}{\arg\min}{\left\|{\hat{\delta B}}_{\text{ref}}\left(r_{\text{in}},\theta \right)-\left(\frac{\Delta \chi }{6}\left(3\;\cos^{2}\;\theta -1\right)B_{0}+c\right)\right\|}_{2}. \end{array}$$

Fitting *c* effectively amounts to shifting the midline of the data to match the model (across all angles). We used a bisquare-weighting method to fit this midline. We include a few examples of $\hat{\Delta \chi }$ estimation using equation (6) in Table S.1.

We apply this frequency referencing method after each phase estimation algorithm. To investigate repeatability with respect to the location of frequency reference estimate, we apply this process in 13 different regions selected across static areas of the phantom (Figure 2(a)).

### 2.5. Error Analysis

We use the theoretically determined values of Δ*χ* to predict the field values at each angle that would have been measured with ideal methods in Steps 1 and 2. We then compute the error (Hz) between measured frequency $\;{\hat{\delta B}}_{\text{ref}}\left(r_{\text{in}},\theta \right)$ and expected frequency offset:

(7) $$\begin{array}{c} ɛ\left(r_{\text{in}},\theta \right)=\;{\hat{\delta B}}_{\text{ref}}\left(r_{\text{in}},\theta \right)-\left(\frac{\Delta \chi_{\text{true}}}{6}\left(3\;\cos^{2}\;\theta -1\right)B_{0}+c^{*}\right), \end{array}$$

where *c*^*∗*^is obtained by solving equation (6) with Δ*χ* set to Δ*χ*_true_.

To account for any dependence on tube content, we compute the absolute relative error:

(8) $$\begin{array}{c} \epsilon_{r}\left(r_{in},\theta \right)=\frac{\left|\epsilon \left(r_{in},\theta \right)\right|}{B_{0}\Delta \chi }, \end{array}$$

where the absolute value is used instead of the signed error due to the irrelevance of sign in this context.

Error statistics were computed for each voxel in the tube ROI (mean tube ROI sizes were 17 pixels at 1 mm resolution and 57 pixels at 0.5 mm resolution), for each slice (2 slices per tube), each tube (5 total), each angle (9 total rotations), using each applicable phase estimation method (from a possible 10), each background phase removal ROI (13 total), and each sequence with its respective resolution and TR variations (11 total). Cumulatively, 2.36 million frequency values were analyzed in this experiment.

The large number of data points allows us to extract statistics about *ε* and *ε*_*r*_ including their probability mass function, Pr(*ε*) and Pr(*ε*_*r*_). The probability of *ε* can exhibit a multimodal distribution and therefore is not a Gaussian. While we can report the absolute bias and standard deviations from such a distribution, it would not be descriptive of Pr(*ε*). A more practical measure is the likelihood of observing absolute relative errors less than or equal to a threshold, *τ*. This is obtained by integrating Pr(*ε*_*r*_) between 0 and *τ* a measure known as the Cumulative Distribution Function (CDF),

(9) $$\begin{array}{c} F_{\varepsilon_{r}}\left(\tau \right)=\;\mathrm{Pr}\left(\varepsilon_{r}\leq \;\tau \right). \end{array}$$

The CDF can be used to capture phase errors that are dominated by outliers, as well as phase errors that result from generally poor/unreliable model fitting. The ideal CDF is a step function, and any presence of outliers/large errors yields a CDF with slow convergence to 1. The frequency of large errors is seen from the magnitude of the deviation of the CDF from 1.0 at any given threshold.

## 3. Results

Figure 2(a) shows a magnitude GRE image of tube 3 (0.5 mM GdCl_3_) in one orientation.  Figure 2(b) shows frequency offset images corresponding to different rotations.  Figure 2(c) shows a typical field difference between two angles (*δB*(*r*, *θ*_2_) −  *δB*(*r*, *θ*_1_)). According to equation (3), this difference is equal to *δB*_Δ*χ*_(*r*, *θ*_2_) − *δB*_Δ*χ*_(*r*, *θ*_1_)+*δB*_0_^*rot*^(*θ*_2_) − *δB*_0_^*rot*^(*θ*_1_), predicting spatial variations only in locations close to areas with susceptibility changes and a constant field in homogeneous locations. This is precisely what we observe in Figure 2(c).  Figure 2(d) shows a plot of the frequency values for a voxel inside the tube prior to frequency referencing (symbol “*x*”). The resulting plot does not follow the expected sinusoid (solid line). After applying the frequency referencing step, we observe the expected sinusoidal shape (symbol “*o*”).

**Figure 3:**
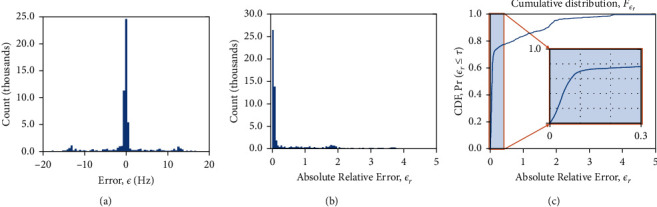
(a) The histogram of the error *ε* (in Hz) when the measurement is obtained with the SEGE + GBC phase estimation method. The error data is pooled over all ROI voxels, all backgrounds, rotations, and tubes. (b) The histogram of the absolute relative error *ε*_**r**_ seen with the same measurement. (c) The corresponding Cumulative Distribution Function **F**_*ϵ*_**r**__of the error in (b). The inset figure shows a portion of this CDF within [0, 0.3] to better illustrate the distributions within reasonable error range. In this case, the CDF shows that the probability of obtaining *ε*_**r**_ less than 0.1 is around 0.66 with the SEGE + GBC pair. The maximum observed *ϵ* was 27.0 Hz, and the maximum observed *ε*_**r**_ was 6.85, illustrating the occasional large errors that may result with SEGE + GBC.

Figure 3 shows the resulting histogram of frequency errors and resulting CDF. From Figures 3(a) and 3(b), we note the probability of *ε* for this example exhibits a multimodal distribution. We can see from the CDF in Figure 3(c) that the probability of observing errors less than 10% (*F*_*ε*_*r*__(0.1)) is about 66%.

**Figure 4:**
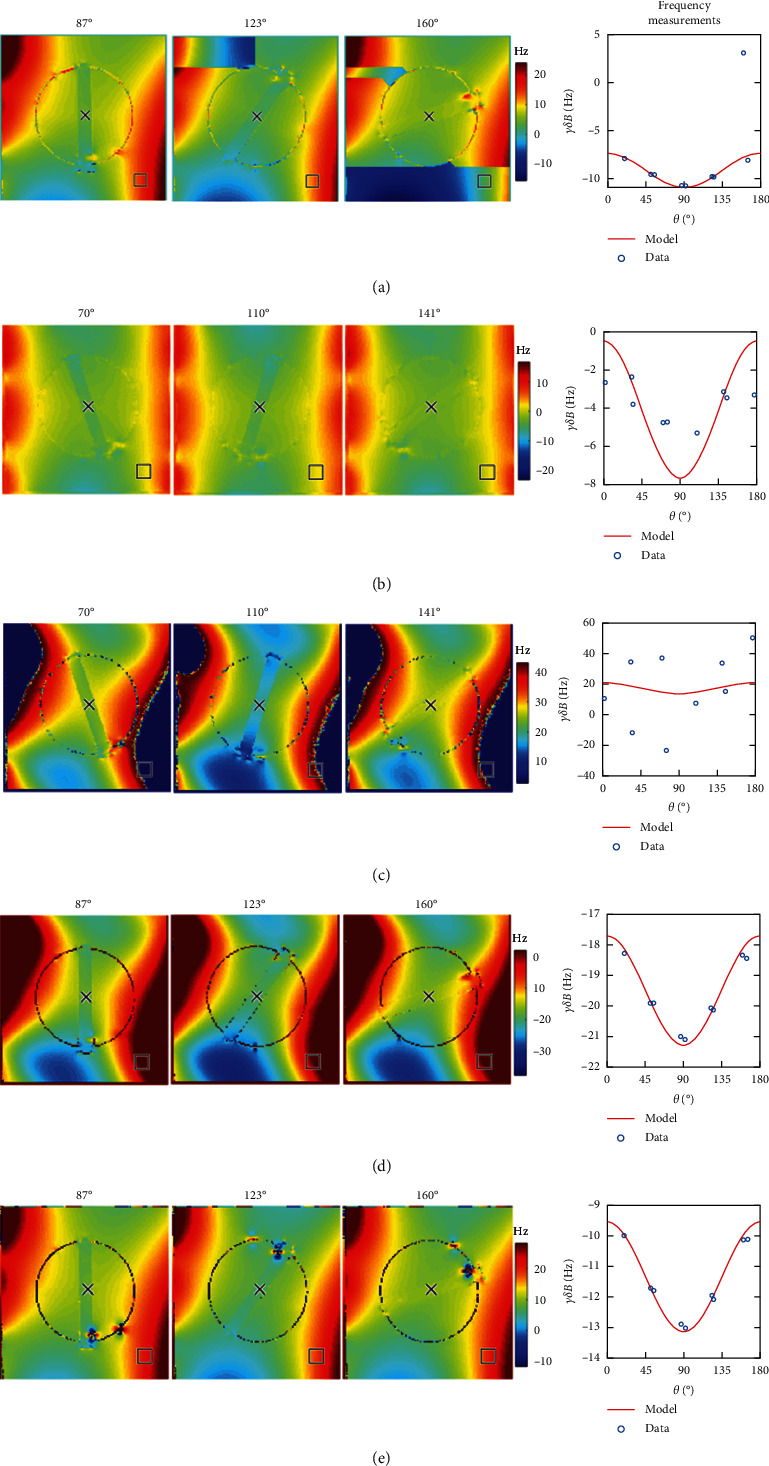
Each row in this figure shows five different examples of sequence (at 1 mm resolution, TR = 45 ms) and phase estimation method pairs for three different angles of rotation in different tubes. These examples were selected to illustrate the spatial nature of different phase estimations errors and artifacts. The corresponding plots in the last column show the resulting frequency measurements as a function of angle of rotation (modulo 180°), for a voxel inside the tube, after frequency referencing (blue circle). The predicted frequency offset as obtained from equation (1) is also shown in solid red line. The background ROI (#9) used is shown with a square overlaid on the frequency maps. (a) SEGE + GBC shows a phase wrapping error in tube 3 in the frequency reference ROI for the 160-degree angle. (b) SEGE + Laplace shows smoothly varying frequency maps; however, the values deviate from the expected result at every angle. (c) MEGE + Slope, without 3D phase unwrapping, shows the presence of phase wrapping in areas with large frequency values. The frequency reference ROI is within a phase wrapped region across all angles. (d, e) MEGE + MAGPI-unopt and MAGPI's phase estimation show frequency maps consistent with values predicted from the model.

**Figure 5:**
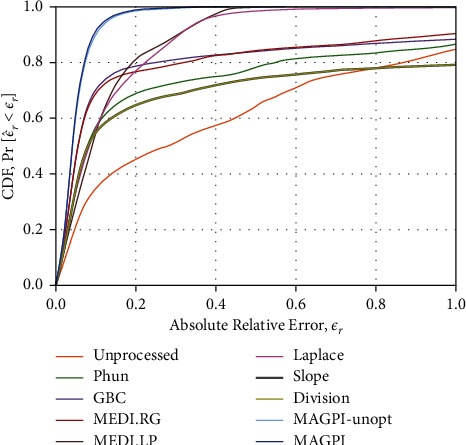
CDF **F**_*ε*_**r**__of the absolute relative error obtained for each of the phase estimation methods studied in this work, when the error is pooled over all sequences, slices, tubes, angles, and frequency referencing ROIs. Note that some methods do not converge to probability of 1, due to the presence of errors greater than 100%.

**Figure 6:**
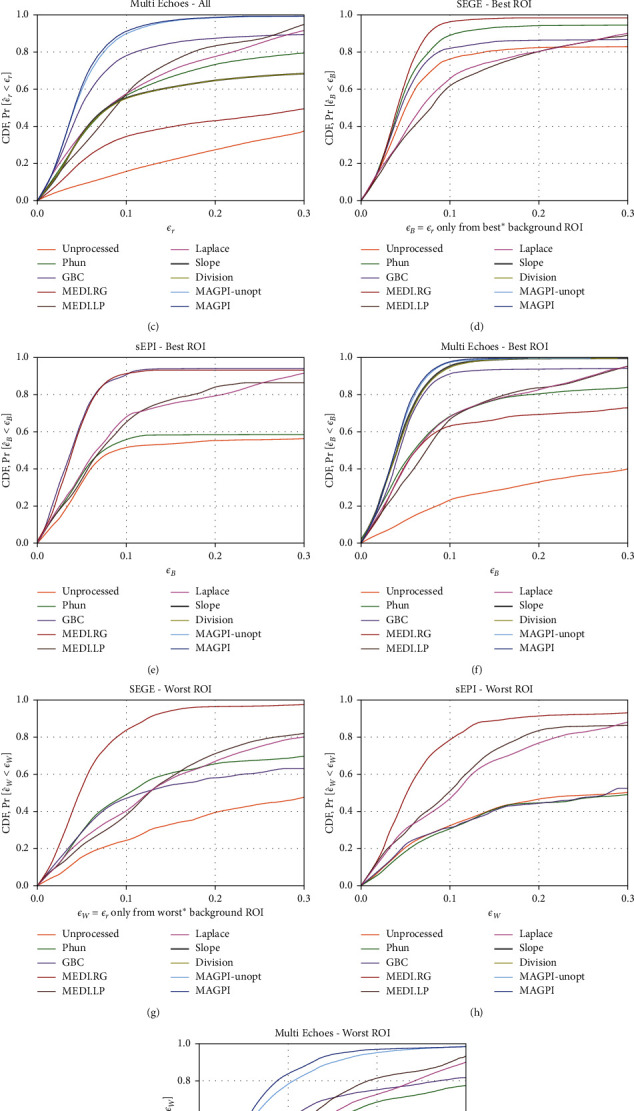
The CDFs for each acquisition and phase estimation method pair. Each column corresponds to a different type of acquisition: single-echo acquisitions (SEGE) are shown in the first column, accelerated acquisitions with sEPI in the second, and multiecho acquisitions (MEGE and MAGPI) in the third. The first row (a–c) shows the CDF when all the error data is pooled, over all voxels, slices, tubes, and background ROIs. Given the large variability in performance for different background ROIs, we show in the second (d–f) and third (g–i) rows the CDFs obtained in the ROIs with the maximum and minimum **F**_*ϵ*_**r**__(0.1), respectively. A highly reproducible method will have a similar curve shape across all plots.

Table 3 summarizes the error statistics for all combination of sequences and algorithms. The first column lists the sequence type, and the second and third columns indicate the name and category of each postprocessing algorithm, respectively. We report the mean and standard deviations of both *ε* and *ε*_*r*_. We also report *F*_*ϵ*_*r*__(0.1) pooled over all background ROIs, as well as the range (minimum, *F*_*ε*_*rW*__(0.1), and maximum, *F*_*ε*_*rB*__(0.1)) of *F*_*ε*_*r*__(0.1) encountered in those ROIs. These quantities are computed from data that includes all tubes and angles. A representative subset of these results is selected for more detailed analysis and illustration in Figures 4–6.

Figure 4 shows a subset of frequency-offset images for different sequence + algorithm pairs, at 3 of the 9 angles of rotation. This figure illustrates typical challenges with phase estimation methods. For example, in Figure 4(a), we observe phase unwrapping errors in SEGE + GBC, with abrupt jumps across contiguous regions. The corresponding frequency versus angle plot (last column in Figure 4(a)) shows that incorrect frequency referencing in these areas (square in figure) yields occasional mismatch between measurement and predictions at certain angles. SEGE + Laplace demonstrates a smoothly varying frequency map across the FOV; however, the resulting data deviates from the expected theoretical values at almost every angle (Figure 4(b)). MEGE + Slope, a direct temporal phase estimation method (MEGE category 3), exhibits phase wrapping errors when the underlying frequency value is larger than the bandwidth allowable by ΔTE(Figure 4(c)). Placing a frequency referencing ROI in these areas yields incorrect values at the respective angles. Note that the example frequency reference ROI (#9 in Figure 2) is meant to highlight the phase errors or artifacts observed. Figures 4(d) and 4(e) show that the results from MAGPI-unopt and MAGPI are consistent with those predicted from theory.

The CDF of *ε*_*r*_ collects the errors, such as those observed in Figure 4, over a variety of acquisition and processing parameters.  Figure 5 shows *F*_*ε*_*r*__(*τ*) for all algorithms when *ε*_*r*_ is pooled over all voxels, background ROIs, slices, tubes, and sequence variations. This represents an overall summary of algorithm behavior, irrespective of which parameter was used in acquisition and postprocessing. We see that MAGPI attains a nearly ideal CDF, with 0.91 probability of relative errors, *ε*_*r*_, less than 0.1 (*F*_*ε*_*r*__(0.1)=0.91) and rapidly converges to 1 (Figure 5). MAGPI and MAGPI-unopt achieve similar CDFs, with MAGPI performing slightly better, as expected. MEDI.RG and GBC phase unwrapping methods, both based on region growing, have similar CDFs, with *F*_*ε*_*r*__(0.1)=0.69 and *F*_*ε*_*r*__(0.1)=0.70, respectively. The unprocessed phase images have the most artifacts and, thus, the lowest CDF across all *ε*_*r*_.  Figure 5 focuses on the CDF for *ε*_*r*_ in [0, 1.0] to highlight the different convergence pattern (distribution/frequency of errors) in that domain. The CDF extends beyond *ε*_*r*_=1.0 for any occurrence of relative errors greater than 100%.

Next, we explore the behavior of *F*_*ε*_*r*__(*τ*) as a function of data acquisition strategy. In Figures 6(a)–6(c), we group results by three sequence types: SEGE, sEPI, and MEGE. Since MAGPI is a multiecho sequence, we include MAGPI in the MEGE category. For each CDF curve *F*_*ϵ*_*r*__(*τ*), *ϵ*_*r*_ is pooled across all pixels, background ROIs, slices, tubes, and variations of TR and resolution within that sequence type. We also explore variability of *F*_*ϵ*_*r*__(*τ*) with the frequency referencing method. For each sequence, we show CDFs in the ROI with the maximum *F*_*ϵ*_*r*__(*τ*=0.1) (*F*_*ϵ*_*rB*__, Figures 6(d)–6(f)) and the ROI with the minimum *F*_*ϵ*_*r*__(*τ*=0.1) (*F*_*ϵ*_*rW*__, Figures 6(g)–6(i)) for a given data set. The separation between *F*_*ϵ*_*rB*__ and *F*_*ϵ*_*rW*__demonstrates the robustness (or lack thereof) of a method to frequency reference ROI selection.

Table 4 shows the dependence of the CDF on scan variability and tube contents. Because the performance of some methods is dominated by frequency reference ROI (seen in Table 3 and Figure 6), Table 4 shows results for the largest **F**_*ϵ*_**r**__(10%) for a given sequence + method pair observed over all frequency reference ROIs, for every method, sequence, scan variation, tube, and slice.

## 4. Discussion

A reliable pulse sequence protocol, with repeatable and reproducible phase estimation, is a necessary step to develop robust QSM methods for clinical use. Previous work examined the reproducibility of certain QSM methods using phantoms [22, 25, 55], simulation [22, 25, 56], and human subjects [19, 56]. We used a rotating-tube phantom to quantitatively evaluate methods used for data acquisition and phase estimation. This is not the first study using long tubes nor is it the first to position tubes relative to B_0_; however, compared to previous work [22, 24, 25, 55, 57–59], more acquisition and phase estimation methods were considered. Specifically, we used ∼90 different combinations of pulse sequences and phase estimation methods to analyze millions of measurements from different ROIs, tube contents, rotations, and sequence parameters. This vast amount of data ultimately allowed us to estimate the probability distribution of phase error with every QSM method, along with other important statistics. Additional phase estimation methods could be retrospectively used on the data set, which we aim to make publicly available.

Our results showed varying degrees of accuracy and precision over all tested methods. For example, while the majority of methods resulted in *μ*_*ϵ*_ less than 1 Hz (note the particularly small *μ*_*ϵ*_ with MEDI.LP, Laplace, MEGE + MAGPI-unopt, and MAGPI), the only methods with *μ*_*ϵ*_*r*__ <10% are SEGE + MEDI.RG (7.6%), MEGE + MAGPI-unopt (5.3%), and MAGPI (5.0%). We observed a similar trend with precision, whereby methods with the lowest relative standard deviation *σ*_*ϵ*_*r*__ were MEGE + MEDI.LP (8.9%), MEGE + MAGPI-unopt (4.8%), and MAGPI (4.1%). The detailed behavior of the error is captured by the CDF (or PDF) of the data (Figure 5). A summary of the CDF is in the second-to-last column of Table 3 where we show the probability of observing relative errors <10%, *F*_*ϵ*_*r*__(10%), which captures the frequency by which *relatively* acceptable errors occur. An advantage of the Laplace-based methods is that they had smooth phase maps with qualitatively no apparent phase jumps. However, analysis showed that Laplace phase images result in quantitatively larger errors (a low *F*_*ϵ*_*r*__(10%)) than other methods, suggesting incorrect phase unwrapping results, similar to the work of Chen et al. [60]. Other methods with low *F*_*ϵ*_*r*__(10%) were the unprocessed phase data, MEGE + Slope/Div, and MEGE + MEDI.RG, which had large phase unwrapping errors in a significant proportion of the data. *F*_*ϵ*_*r*__(10%) is an arbitrary point at which we highlight the behavior of *F*_*ϵ*_*r*__ and does not represent the entirety of the distribution of error (or CDF). For example, *F*_*ϵ*_*r*__(10%) of SEGE + MEDI.RG was comparable to MEGE + MAGPI-unopt and MAGPI, despite the comparatively poorer (larger) *μ*_*ϵ*_*r*__ and *σ*_*ϵ*_*r*__ of SEGE + MEDI.RG. This is due to relative errors falling mostly within the chosen 10% threshold for these methods.

We explored the dependence of errors on frequency reference ROI location (Figure 6). Since phase estimation errors (particularly large errors) are undesirable anywhere in the FOV, any spatial variation of the CDF highlights the potential dependence of the method on user intervention and/or its automated processing. We show the range of *F*_*ϵ*_*r*__(10%) observed across the 13 different frequency reference ROIs in the last column of Table 3. The results suggest that the most repeatable methods across background ROIs are SEGE + MEDI.RG, sEPI + MEDI.RG, MEGE + MAGPI-unopt, and MAGPI.

The MEGE data was processed using four broad classes of postprocessing algorithms. We note the following about these algorithms:

- The results obtained with methods in Categories 1 and 2 were fundamentally similar. That is, additional 1D-temporal processing does not alter the performance of 3D spatial unwrapping methods (with the exception of Laplacian-based methods, which we discuss below). This redundancy is due to the inherent Nyquist limitation associated with the echo spacing. As a result, we focus on the distinctive results of Category 1: spatial phase unwrapping + weighted averaging of echoes (Table 3).
- Some postprocessing methods used in MEGE Categories 1 and 2 performed more poorly with MEGE than with SEGE (e.g., MEDI.RG). We believe this is due to the relatively larger bandwidth used with MEGE acquisitions, resulting in noisier images at each echo. Higher BW acquisitions were needed with MEGE to accommodate temporal methods (MEGE Categories 3 and 4), which require short echo spacing. It is possible that MEGE + MEDI.RG would perform better with lower BW (wider echo spacing). Due to time/complexity constraints, we were unable to explore every possible MEGE variation that favors specific algorithms. This is a limitation of this study.
- MEGE Category 3 methods (Division/Slope) were straightforward to apply but resulted in a wide range of errors. This is due to large errors observed in frequency reference ROIs where the underlying frequency-offset value is larger than what is allowable by the smallest echo spacing. While such errors are avoidable with shorter echo spacing, this is not always possible (as was the case here) due to hardware constraints on readout bandwidth, resolution, FOV, and so forth. While Slope and Division are straightforward to apply, they result in a suboptimal combination of echoes, with noisy phase estimates.
- MEGE Category 4 methods have a similar performance to that of Category 3 methods. That is, spatial phase unwrapping did not seem to markedly improve the performance of temporal phase unwrapping. This is potentially due to the hard-to-unwrap noisy boundary lines observed with Category 3 methods, as shown in MEGE + Slope example in Figure 4, which are still present with Category 4 methods.

Finally, we explored the dependence of the CDF on scan variability and tube contents (Table 4), and we note the following:

1. Not all sequence + method pairs were invariant to scan and/or tube content. sEPI + Phun and sEPI + Unprocessed had greatest variability, followed by all of the Laplace-based methods (LP, MEDI.LP), indicating that some methods produced inconsistent phase even in their best-case scenario. Laplace-based techniques generated smoothly varying, though numerically inaccurate phase maps, irrespective of sequence, and sEPI + Phun suffered from sequence-dependent errors.
2. Considering the best-case ROI scenario, we did not observe consistent performance differences between pairing methods with either SEGE or sEPI. The sEPI sequence provides a significant acceleration over SEGE via its segmented GRE approach and performed better with some methods (GBC) and worse with others (Phun).
3. Table 4 compares Sequence + Method pair performance as a function of variability of scan parameters. For example, MEGE + Slope performed consistently well (*F*_*ϵ*_*rB*__(10%) >  0.95) irrespective of the scan type used, while SEGE + Laplace was not always able to estimate the correct phase, irrespective of scan. SEGE + GBC, though, only struggled with Scan 2, which had a shorter TR than Scan 1.
4. The same analysis can be applied to tube contents. MAGPI achieved *F*_*ϵ*_*rB*__(10%) close to 1.0 for all tubes and *F*_*ϵ*_*rB*__(10%) = 0.91 in Tube 5. sEPI + Phun had inconsistent performance across tubes of similar content (Tubes 1, 2, 3, and 4). Among the methods that maximized *F*_*ϵ*_*r*__(0.1), Tube 5 exhibited lower *F*_*ϵ*_*rB*__(10%) compared to other tubes, which could be due to challenges defining Δ*χ*_th_ and the complexity of CuSO_4_ compared to GdCl_3_.

Overall, the consistently good performance across tube content, scan, and rotation angle in the absence of phase estimation errors validates the Δ*χ* model (equation (1)) and demonstrates that the rotating-tube phantom itself did not introduce unexpected detrimental effects to the phase measurements. In previous work, QSM performance improved with higher isotropic spatial resolution and higher coverage [56, 61]. Here, slice thickness was 2.0 mm for all scans, which may explain why performance did not drastically change with resolution. Additionally, Zhou et al. [61] and Karsa et al. [56] examined the entire QSM process, including inversion, which was not addressed here. Olsson et al. [22] used a phantom with tubes of Gd in comparable concentrations, though only one tube was used when varying angle with respect to *B*_0_ (five angles). That study used one method to estimate QSM [62–64] over multiple spatial resolutions, volumes, and inversion parameters. Similar to the work of Karsa et al. [56], Olsson et al.'s results improved with increasing resolution and volume coverage, and, similar to our results, Olsson et al. observed errors in phase estimation using [47], compared to the theoretical result.

A limitation of this work is that the geometric structure of the phantom was not identical to that encountered *in vivo*. *In vivo* imaging may present different sources of phase errors not included here (e.g., eddy currents, susceptibility-induced signal drops), and the phantom could present some challenges that are not encountered or are less prominent in the brain. This is a common problem with nearly all phantom studies, and it is counterbalanced by the advantage of having a known truth, which is not possible *in vivo*. Errors observed in phantoms are frequently observed *in vivo*, even when the geometry of the phantom is a gross simplification of human anatomy. For example, in T_1_ estimation, while phantoms can be used to refine a method, errors are amplified when methods are applied *in vivo* [65]. The many parameters, sequences, and processing steps considered here are useful to evaluate the robustness of phase estimation techniques, and this work can be viewed as a complement to other efforts seeking to evaluate the accuracy of QSM techniques, such as the use of simulated data [66, 67].

We introduced a wide range of variability to test repeatability and reproducibility of many data acquisition scenarios. While the performance of each method could be improved with additional “intervention” and potentially adapting the acquisition parameters to the intended postprocessing methods to be used later, our intent was to assess the ability of existing techniques in diverse imaging scenarios encountered in reality. The degree to which Steps 3 and 4 of QSM are sensitive to the errors introduced in Steps 1 and 2 requires further investigation. This phantom validation study allowed us to set a quantitative limit on the performance of various Steps 1 and 2 methods. Ongoing work focuses on evaluating the performance of a subset of these methods, paired with Steps 3 and 4 methods.

## 5. Conclusion

In this work, we used a rotating-tube phantom to explore sources of error in QSM data acquisition and phase estimation. To assess the robustness and repeatability of methods, we did not manually intervene. The two most impactful parameters on reproducibility of measurements were (a) acquisition protocol (e.g., single echo or multiple echoes) and (b) phase errors. The most repeatable and reproducible approaches were MAGPI and MAGPI-unopt, both methods based on the maximum-likelihood approach in phase estimation. For the remaining methods, performance varied greatly, even when systematically applied to the same underlying data from the same sequence or with the same method across different sequences. This assessment of which methods are repeatable and reproducible without manual intervention is an important step towards using QSM pipelines in clinical settings without experienced users.

## Figures and Tables

**Table 1:** All data acquisition parameters.

Sequence	Scan ID	TR (ms)	Echo times (ms)	Bandwidth (Hz)	Alpha (deg)	Resolution (mm^3^)	Acquisition time (min:s)
Single-echo GRE (SEGE)	1	25	16	80	15	1.0 × 1.0 × 2.0	1 : 59
	2	35	25.7	80	15	0.5 × 0.5 × 2.0	4 : 35
	3	45	30	80	15	1.0 × 1.0 × 2.0	3 : 35

Multiecho GRE (MEGE)	1	25	2.5, 6.2, 9.9, 13.6, 17.3, 21.0	500	15	1.0 × 1.0 × 2.0	1 : 59
	2	35	3, 7.8, 12.6, 17.4, 22.2, 27.0	530	15	0.5 × 0.5 × 2.0	4 : 35
	3	45	2.5, 6.2, 9.9, 13.6, 17.3, 21.0, 24.7, 28.4, 32.1, 35.7, 39.5	500	15	1.0 × 1.0 × 2.0	3 : 35

sEPI	1	72	27, ETL = 15	430	20	1.0 × 1.0 × 2.0	0 : 31
	2	72	27, ETL = 15	430	20	0.5 × 0.5 × 2.0	0 : 52

MAGPI	1	25	4.1, 8.9, 12.6, 16.3, 20.0	190	15	1.0 × 1.0 × 2.0	1 : 59
	2	35	7.6, 13.0, 17.3, 21.5, 25.7, 30.0	250	15	0.5 × 0.5 × 2.0	4 : 35
	3	45	6.6, 13.0, 18.5, 23.8, 29.0, 34.2, 39.4	200	15	1.0 × 1.0 × 2.0	3 : 35

**Table 2:** All phase estimation methods. The first six methods were combined with other common phase processing techniques to process the multiecho data, as described in the Methods section.

Sequence	Method	Summary
SEGE, MEGE, sEPI	Unprocessed	Default channel combined output phase image produced by the scanner
	Phun	Region-based algorithm [46]
	GBC	Goldstein's branch cut method [42]
	MEDI.RG	Region growing algorithm from MEDI Toolbox [45, 47]
	MEDI.LP	Laplacian algorithm from MEDI Toolbox [44, 45]
	Laplace	Laplacian algorithm [40]

MEGE	Slope	Computes the frequency offset from the slope of the complex data across echoes [41]
	Division	Computes the frequency offset from the complex division of the data at successive echoes [43]
	MAGPI-unopt	Applies the MAGPI postprocessing algorithm using a random (unoptimized) subset of echoes from the MEGE sequence. Since MAGPI requires uneven echo spacing, only four unequally spaced echoes were selected [37]

MAGPI	MAGPI	Maximum-likelihood estimate of phase from optimal echo spacing [37]

**Table 3:** Error statistics for each of the estimation methods and acquisition protocols. The mean and standard deviation of error measurements *ε* (Hz), as well as the absolute relative errors *ε*_*r*_, are provided in columns 4–7. Columns 8-9 show *F*_*ϵ*_*r*__(0.1) of the overall data, as well as the range (min and max) of *F*_*ϵ*_*r*__(0.1) across reference backgrounds.

Protocol	Postprocessing	Method	*μ*_*ϵ*_(Hz)	*σ*_*ϵ*_ (Hz)	*μ*_*ϵ*_*r*__	*σ*_*ϵ*_*r*__	**F**_*ϵ*_**r**__(0.1)	[**F**_*ϵ*_**rW**__(0.1), **F**_*ϵ*_**rB**__(0.1)]
SEGE	Spatial 3D unwrap	Unprocessed	0.29	3.85	0.52	0.92	0.48	[0.24, 0.76]
		Phun	0.21	5.12	0.35	1.38	0.67	[0.49, 0.89]
		GBC	−0.22	4.83	0.53	1.08	0.66	[0.47, 0.82]
		MEDI.RG	−0.19	1.60	0.08	0.22	0.90	[0.84, 0.96]
		MEDI.LP	−0.01	1.07	0.14	0.12	0.50	[0.38, 0.62]
		Laplace	0.00	1.01	0.14	0.15	0.54	[0.40, 0.66]

sEPI	Spatial 3D unwrap	Unprocessed	−0.34	4.19	0.58	0.76	0.43	[0.32, 0.52]
		Phun	−0.10	5.23	0.73	1.30	0.45	[0.31, 0.56]
		GBC	−0.32	3.80	0.42	0.97	0.66	[0.31, 0.91]
		MEDI.RG	1.03	4.14	0.32	1.15	0.85	[0.79, 0.91]
		MEDI.LP	0.00	1.14	0.12	0.12	0.57	[0.51, 0.65]
		Laplace	0.00	0.95	0.12	0.11	0.58	[0.47, 0.68]

Multiecho	1. Spatial 3D unwrap ⟶ temporal average	(i) Unprocessed	0.01	2.60	0.48	0.41	0.16	[0.08, 0.23]
		(ii) Phun	0.39	4.75	0.30	1.23	0.57	[0.48, 0.69]
		(iii) GBC	0.31	2.21	0.15	0.36	0.78	[0.59, 0.91]
		(iv) MEDI.RG	−0.37	3.47	0.66	0.95	0.35	[0.09, 0.63]
		(v) MEDI.LP	−0.01	0.89	0.11	0.09	0.58	[0.49, 0.67]
		(vi) Laplace	0.00	0.82	0.12	0.12	0.58	[0.44, 0.68]
	2. Spatial 3D unwrap ⟶ temporal 1D unwrap ⟶ temporal average	(i) Phun	0.19	7.66	0.94	2.23	0.55	[0.12, 0.96]
		(ii) GBC	0.20	7.78	0.96	2.25	0.55	[0.12, 0.96]
		(iii) MEDI.RG	0.13	7.95	0.97	2.29	0.55	[0.12, 0.96]
		(iv) MEDI.LP	−0.01	0.89	0.11	0.09	0.58	[0.49, 0.67]
		(v) Laplace	0.00	0.82	0.12	0.13	0.58	[0.45, 0.68]
	3. Temporal estimation	(i) Slope	0.26	9.28	1.15	2.79	0.55	[0.13, 0.96]
		(ii) Division	0.26	9.27	1.15	2.78	0.55	[0.13, 0.95]
	4. Temporal estimation ⟶ spatial 3D unwrap	(i) Phun	0.04	11.04	1.17	3.02	0.53	[0.06, 0.95]
		(ii) GBC	0.17	9.18	1.10	2.69	0.54	[0.09, 0.95]
		(iii) MEDI.RG	0.47	62.07	10.11	16.54	0.21	[0.00, 0.76]
		(iv) MEDI.LP	0.16	3.87	0.54	1.16	0.43	[0.20, 0.70]
		(v) Laplace	0.22	6.89	0.89	2.05	0.36	[0.10, 0.66]
	5. MAGPI	(i) MAGPI unopt	0.00	0.30	0.05	0.05	0.90	[0.78, 0.97]
		(ii) MAGPI	0.00	0.27	0.05	0.04	0.91	[0.84, 0.98]

**Table 4:** Probability of relative errors <0.1 in the background ROI that maximizes **F**_*ϵ*_**r**__(0.1) across sequences, scan variability (ID is as listed in Table 1), and phantom tube components. The tube contents are 1.0 mM GdCl_3_ in tubes 1 and 2, 0.5 mM GdCl_3_ in tubes 3 and 4, and 3.2 mM CuSO_4_ in tube 5. The last column is the standard deviation of all *F*_*ϵ*_*rB*__(0.1) for the given method.

Method	Sequence	Scan ID 1	Scan ID 2	Scan ID 3	Tube 1	Tube 2	Tube 3	Tube 4	Tube 5	Std **F**_*ϵ*_**rB**__(0.1)
Unprocessed	SEGE	0.79	0.86	0.47	0.59	0.88	0.83	0.89	0.68	0.32
	MEGE	0.25	0.21	0.27	0.25	0.23	0.25	0.26	0.18	
	sEPI	0.51	0.55	—	1.00	0.00	0.86	0.06	0.77	

Phun	SEGE	0.91	0.96	0.68	0.98	0.88	0.83	0.87	0.88	0.24
	MEGE	0.67	0.73	0.64	0.89	0.51	0.54	0.66	0.80	
	sEPI	0.56	0.58	—	0.92	0.00	0.87	0.37	0.79	

GBC	SEGE	0.96	0.73	0.96	0.75	0.79	0.97	0.93	0.73	0.08
	MEGE	0.95	0.88	0.96	0.89	0.93	0.97	1.00	0.80	
	sEPI	0.89	0.97	—	0.95	0.89	0.90	1.00	0.82	

MEDI.RG	SEGE	0.97	0.96	0.97	1.00	0.94	1.00	1.00	0.89	0.20
	MEGE	0.24	0.76	0.65	0.63	0.61	0.64	0.67	0.63	
	sEPI	0.91	0.92	—	1.00	0.84	0.94	0.97	0.84	

MEDI.LP	SEGE	0.63	0.63	0.59	0.36	0.58	0.53	0.96	0.70	0.18
	MEGE	0.68	0.67	0.64	0.36	0.75	0.53	0.95	0.76	
	sEPI	0.65	0.66	—	0.36	0.63	0.53	1.00	0.78	

Laplace	SEGE	0.57	0.68	0.68	0.36	0.97	0.52	0.85	0.56	0.19
	MEGE	0.62	0.72	0.64	0.36	1.00	0.69	0.90	0.46	
	sEPI	0.66	0.74	—	0.40	0.93	0.58	0.89	0.60	

Slope	MEGE	0.96	0.95	0.97	0.99	1.00	0.95	0.99	0.82	0.06

Division	MEGE	0.95	0.94	0.96	0.99	1.00	0.95	0.99	0.78	0.07

MAGPI-unopt	MEGE	0.97	0.97	0.97	1.00	1.00	0.98	1.00	0.87	0.04

MAGPI	MAGPI	0.97	0.98	0.97	1.00	1.00	0.98	1.00	0.91	0.03

## Data Availability

The authors are performing subsequent analysis of the data for an extension of this paper. After completion, the data and code will be made publicly available.

## Abbreviations

BW: Bandwidth
CDF: Cumulative Distribution Function
FOV: Field of view
FR: Frequency referencing
GBC: Goldstein's branch cut
GRE: Gradient echo
LP: Laplace
MAGPI: Maximum AmbiGuity distance for Phase Imaging
MEDI: Morphology enabled dipole inversion
MEGE: Multiecho gradient echo
QSM: Quantitative Susceptibility Mapping
RG: Region growing
ROI: Region of interest
SEGE: Single-echo gradient echo
sEPI: Segmented Echo Planar Imaging.

## Supplementary Materials

Supplementary Materials A supplementary material file is included which provides additional details on the frequency referencing methods (Section S.1) and the determination of reference values (Section S.2).
