# Supplementary material for: Comparison of Phase Estimation Methods for Quantitative Susceptibility Mapping Using a Rotating-Tube Phantom
Source: Radiol Res Pract. 2021 Nov 24;2021:1898461. doi: 10.1155/2021/1898461 (PMC8635951; doi:10.1155/2021/1898461)
Supplement: Supplementary Materials — A supplementary material file is included which provides additional details on the frequency referencing methods (Section S.1) and the determination of reference values (Section S.2). [file 1898461.f1.docx]

# Supplementary Material: Additional Experimental Details

## S.1 Frequency Referencing Methods

The Frequency Referencing method is attempting to remove the frequency offsets due to the rotating apparatus, and not the frequency offset due to susceptibility differences. After the effect of the rotating apparatus is removed, we fit the referenced frequency to the angle of rotation (Eq 1).

We call the process of removing $\delta B_{0}^{rot}\left( \theta\right)$ “frequency referencing”. We compute an estimate of $\delta B_{0}^{rot}\left( \theta\right)$ using the average frequency in a region outside the “Tube + Sphere” system. Our hypothesis is that this yields a good estimate of $\delta B_{0}^{rot}\left( \theta\right)$ for three reasons: first, the region used for referencing (ROIs 1-13 in Fig. 2a) is static and not rotating with the apparatus. Second, the reference region is chosen far enough away from the Tube + Sphere system so that the “local susceptibility effects” (first term in Eq 2) do not affect the accuracy of the estimate of $\delta B_{0}^{rot}\left( \theta\right)$. Finally, since the content in the reference region is homogenous material (water), the average field in each area improves the precision of this estimate.

In the table below, we include a few examples of $\hat{\Delta\chi}$ estimation using this model described in Eq 6.

**Table S.1.** $\hat{\Delta\chi}$ estimation using 1 mm resolution, TR=25 ms imaging data. All reported magnetic susceptibilities are in ppm.

| Tube  Method | 1 | 2 | 3 | 4 | 5 |
| --- | --- | --- | --- | --- | --- |
| ${\Delta\chi}_{theorectial}$ | 0.3362 | 0.3362 | 0.1681 | 0.1681 | 0.0804 |
| Slope $\hat{\Delta\chi}$ | 0.2991 | 0.3177 | 0.1455 | 0.1868 | 0.0649 |
| MAGPI $\hat{\Delta\chi}$ | 0.3157 | 0.2822 | 0.1496 | 0.1930 | 0.0709 |
| GBC $\hat{\Delta\chi}$ | 0.3175 | 0.2985 | 0.1514 | 0.1761 | 0.0605 |
| MEDI.RG $\hat{\Delta\chi}$ | 0.2970 | 0.3270 | 0.1422 | 0.1925 | 0.0595 |

Applying the susceptibility fit, after the frequency referencing step results in a good estimate of $\hat{\Delta\chi}$, as demonstrated by the values in Table S.1.

## S.2 Determination of Reference Values

Susceptibility theory^1^ gives two approaches to determine *Δ*χ_th_ via Curie’s Law: first, using effective permeability, $\mu_{eff}$, based on experimentally determined values from the literature^2^ and second, using the permeability, μ, calculated from the spin orbitals. In the manuscript, we reported the values from the empirically-determined magnetic moment. For GdCl_3_, the theoretically-determined values from both methods were 0.168 ppm and 0.336 ppm, respectively. However, for CuSO_4_, the two theoretical values were 0.0804 ppm, determined using the highest reported Cu^2+^ moment, 2.17 Bohr magnetons, and 0.05112 ppm, determined using the spin orbital angular momentum quantum number, s=1/2, and the Landé g factor=2. We performed independent validation of the theoretical susceptibility values using NMR measurements.

Briefly, NMR susceptibility was determined from the chemical shift or peak separation between a H_2_O reference vial and the sample under test, each in capillaries placed in a standard 5 mm NMR tube. The NMR measurements were made at 14 T (600 MHz) and at 21.42 °C (comparable to the MRI experimental temperature range 21.5 °C +/- 0.5 °C). The measured NMR susceptibility for 0.5 mM GdCl_3_ was 0.176 ppm and for 1.0 mM GdCl_3_ was 0.372 ppm; 5 % and 10 % greater than the theoretical values. The NMR-determined susceptibility for 3.2 mM CuSO_4_ in two separate experiments was 0.075 ppm and 0.0837 ppm. The NMR measurements for CuSO_4_ were 7 % less and 4 % greater than the *Δ*χ_th_ = 0.0804 ppm, and 47 % and 64 % greater than the *Δ*χ_th_ = 0.0511 ppm.

Copper has a low susceptibility value; CuSO_4_ is a more complex solution than GdCl_3_, and as a result, it is useful to test the lower limit of the susceptibility measurements. However, the moment in copper solutions is highly dependent on the local environment, which leads to the broader range of theoretical solutions. Based on the NMR measurements, the empirically-measurement moment is a better representation of the local environment in our experiment (including the CuSO_4_ solution, concentration and tube materials).

*References*

1. Carlin R. Magnetochemistry. Springer; 1986:chap 9.

2. Bain GA, Berry JF. Diamagnetic corrections and Pascal's constants. *J Chem Educ*. Apr 2008;85(4):532-536. doi:DOI 10.1021/ed085p532
